# 4-Repeat Tauopathien – Progressive Supranukleäre Blickparese und kortikobasale Degeneration

**DOI:** 10.1055/a-2895-4480

**Published:** 2026-09-11

**Authors:** Carla Teresa Palleis, Alexander Bernhardt, Günter Höglinger

**Affiliations:** 1 Neurologische Klinik und PoliklinikLMU Klinikum 9183 Ludwig-Maximilians-Universität MünchenMünchenGermany; 2 378588 Deutsches Zentrum für Neurodegenerative Erkrankungen Standort MünchenMünchenGermany; 3 Munich Cluster for Systems Neurology (Synergy)MünchenGermany

**Keywords:** Primäre Tauopathien, Bewegungsstörungen, Atypische Parkinsonsyndrome, Progressive Supranukleäre Blickparese, Kortikobasale Degeneration, atypical Parkinsonism, primary tauopathies, movement disorders, Progressive supranuclear palsy, Corticobasal degeneration

## Abstract

Die Progressive Supranukleäre Blickparese (PSP) und die Kortikobasale Degeneration (Corticobasal Degeneration, CBD) zählen zu den primären Tauopathien, die neuropathologisch durch Ablagerungen von 4-Repeat-Tau-Proteinen charakterisiert sind. Beide Erkrankungen führen zu einer progredienten Beeinträchtigung von Motorik, Gleichgewicht, Sprache, Kognition und Alltagsfunktion. Während die PSP häufig mit einer axial betonten Symptomatik und einer charakteristischen Mittelhirnatrophie einhergeht, zeigt sich bei der CBD meist eine asymmetrische frontoparietale Atrophie mit kortikalen und subkortikalen Defiziten. Aufgrund überlappender klinischer Phänotypen gestaltet sich die Abgrenzung beider Erkrankungen von anderen neurodegenerativen Erkrankungen im klinischen Alltag oftmals schwierig. Dabei ist zwischen der neuropathologisch definierten CBD und dem klinischen Phänotyp des kortikobasalen Syndroms (Corticobasal Syndrome, CBS) zu unterscheiden, welches auch durch andere Pathologien, etwa eine Alzheimer-Pathologie verursacht sein kann. Die Diagnose einer PSP oder CBD erfolgt gemäß den aktuellen klinischen Kriterien und basiert auf der Beurteilung mehrerer funktioneller Domänen entsprechend der Ausprägung der Symptome. Eine krankheitsmodifizierende Therapie steht bislang nicht zur Verfügung. Die Behandlung verfolgt derzeit symptomorientierte Ansätze mit dem Ziel, Lebensqualität und Selbstständigkeit der betroffenen Personen möglichst lange zu erhalten.Dieser Artikel bietet einen aktuellen Überblick über die Diagnose, den Behandlungsansatz, die klinischen Erscheinungsbilder sowie die Behandlungsmöglichkeiten für betroffene Personen mit PSP und CBD.

## Abstract (en)

Progressive supranuclear palsy (PSP) and corticobasal degeneration (CBD) are primary tauopathies that are neuropathologically characterized by deposits of 4-repeat tau proteins. Both diseases lead to a progressive impairment of motor skills, balance, speech, cognition, and everyday functioning. While PSP is often associated with axially emphasized symptoms and characteristic midbrain atrophy, CBD usually presents with asymmetric frontoparietal atrophy with cortical and subcortical deficits. Due to overlapping clinical phenotypes, it is often difficult to distinguish the two diseases from other neurodegenerative diseases in everyday clinical practice. A distinction must be made between neuropathologically defined CBD and the clinical phenotype of corticobasal syndrome (CBS), which can also be caused by other pathologies, such as Alzheimer's disease. The diagnosis of PSP or CBD is made according to current clinical criteria, based on the evaluation of multiple functional domains and the severity of presenting symptoms. Disease-modifying therapies are not yet available. Treatment currently follows symptom-oriented approaches with the aim of maintaining the quality of life and independence of patients for as long as possible. This article provides an up-to-date overview of the diagnosis, clinical presentation, management strategies, and treatment options for individuals affected by PSP and CBD.

## Biologisch und neuropathologisch basierte Krankheitsdefinition

**Abb. 1:**
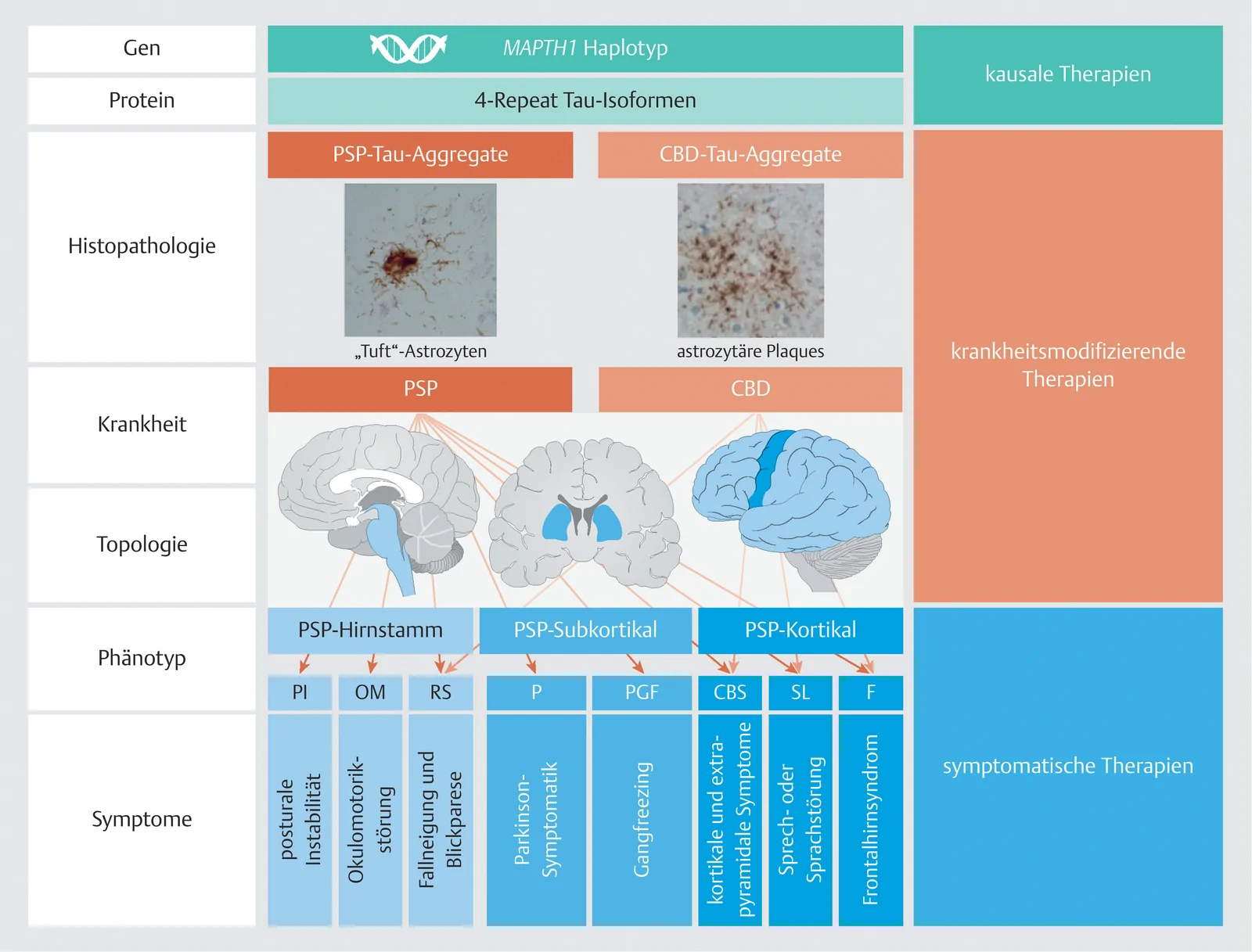
Pathophysiologisches Konzept der progressiven supranukleären Blickparese (PSP) und der kortikobasalen Degeneration (Corticobasal Degeneration, CBD). Homozygotie für den MAPT-H1-Haplotyp prädisponiert für eine erhöhte Expression von aggregationsanfälligen 4-Repeat-Tau-Isoformen. Dennoch sind beides seltene sporadische Erkrankungen ohne mendelische Vererbbarkeit. Tauaggregate scheinen die Tau-Pathologie von Zelle zu Zelle zu verbreiten und krankheitsspezifische zytopathologische Aggregationsmuster zu prägen. “Tuft”(Büschel)-Astrozyten mit Tau-Aggregaten (AT8-Tau-immunreaktiv) in den distalen Astrozyten-Fortsätzen und Komma- oder Kugel-förmige Ablagerungen („Coiled bodies“) in Oligodendrozyten neben neuronalen Fibrillenbündeln („Neurofibrillary Tangles“, NFTs) kennzeichnen die PSP-Pathologie. Astrozytäre Plaques, d. h. ringförmige Ansammlungen kurzer Tau-Aggregate in den proximalen Astrozyten-Fortsätzen, sind die charakteristische astrozytäre Läsion bei CBD (AT8-Tau-Immunfärbung; mit freundlicher Genehmigung von Dr. Sigrun Roeber, München). Während die PSP-Pathologie am häufigsten mit frontal-kortikaler und Hirnstamm-betonter (v. a. mesencephaler) Dominanz beobachtet wird, treten auch kortikale oder transitionale Muster auf. Umgekehrt tritt die CBD-Pathologie am häufigsten in parietal kortikalen und subkortikalen (basalganglionären) Mustern auf. Entsprechend dem anatomischen Muster weisen PSP und CBD ein breites Spektrum klinischer Syndrome auf, darunter progredientes Gangfreezing (PGF), prädominante Störung der Okulomotorik (OM), prädominante Parkinson-Symptomatik (P), prädominante posturale Instabilität (PI), Richardson-Syndrom (RS), kortikobasales Syndrom (Corticobasal Syndrome, CBS), Sprechapraxie oder nicht-flüssige agrammatische Variante der primären progressiven Aphasie (SL, für engl. Speech/Language) und frontales kognitives und Verhaltens-Syndrom (F) oder posteriore kortikale Atrophie (PCA). Die symptomatische Therapie zielt auf die klinischen Syndrome ab. Eine krankheitsmodifizierende Therapie könnte durch Eingriffe in die Mechanismen erreicht werden, die an der Ausbreitung der Pathologie beteiligt sind. Eine kausale Therapie, die darauf abzielt, die Krankheit zu verhindern oder zu heilen, könnte durch Eingriffe in die primären, d. h. die am weitesten vorgelagerten Krankheitsmechanismen möglich sein. Modifiziert nach Höglinger et al 1 .

Die PSP und die CBD sind beides histopathologisch definierte Entitäten. Sie gehören zu den primären Tauopathien mit prädominanter Ablagerung von 4-repeat (4R) Tau-Isoformen. Abb. 1 bietet eine Übersicht über Pathophysiologie, Krankheitsdefinition und Syndrome der primären Tauopathien. Neuropathologisch sind für PSP “Tuft”-(Büschel-)-Astrozyten ( Abb. 1 ) und rundliche Neurofibrillenbündel typisch, für CBD astrozytäre Plaques und ballonierte Neurone 1 . Bei PSP finden sich die meisten Tau-Ablagerungen in den Basalganglien und im Hirnstamm, während bei CBD häufiger auch Ablagerungen im Kortex zu finden sind. Im Gegensatz zur Alzheimer Krankheit (AK) finden sich bei 4R-Tauopathien keine Ablagerungen von β-Amyloid und keine gemischten 3-Repeat/4-Repeat-Tau-Ablagerungen.

## Epidemiologie

Die Prävalenz der PSP wird auf 5–6 pro 100 000 Einwohner 2 und die der CBD auf etwa 1 pro 100 000 Einwohner 3 geschätzt. PSP und CBD treten bei beiden Geschlechtern etwa gleich häufig auf. Das mittlere Erkrankungsalter liegt in der sechsten bis siebten Lebensdekade. Die Zeitspanne von Erstmanifestation bis zur Erstdiagnose beträgt im Schnitt drei bis vier Jahre und das mittlere Überleben etwa sechs bis neun Jahre 4 . Methodische Heterogenität, Fehlklassifikation (klinische vs. pathologische Diagnose) und regionale Unterschiede begrenzen jedoch die Präzision der epidemiologischen Schätzungen. Da der Goldstandard der Diagnose auf der histopathologischen Untersuchung post-mortem fußt und valide *in vivo* Biomarker aktuell noch fehlen, muss von einer höheren Dunkelziffer aufgrund von Unsicherheiten in der klinischen Diagnose ausgegangen werden.

## Ätiopathogenese

4R-Tauopathien treten meist sporadisch auf und es wird eine multifaktorielle Genese aus genetischen, epigenetischen und Umweltfaktoren vermutet, wobei der genaue Krankheitsmechanismus Gegenstand intensiver Forschung ist 5 . Fehlfaltung und Aggregation der 4R-Isoformen des Tau-Proteins führen zu einer irreversiblen Neurodegeneration. Es gibt zudem Hinweise darauf, dass eine mitochondriale Dysfunktion, Störungen der zellulären proteostatischen Mechanismen, neuroinflammatorische Prozesse und ein Prion-artiges Ausbreiten von 4R-Aggregaten entlang der Axone im Gehirn eine Rolle spielen. Für die PSP gibt es eine histopathologische Klassifikation der Verteilung von Tau-Ablagerungen über das Gehirn in sechs konsekutiven Stadien, von den Basalganglien ausgehend über weitere subkortikale und schließlich kortikale Areale 6 . Diese topographische Progression legt einen stereotypen, möglicherweise transneuronalen Ausbreitungsmechanismus der Tau-Pathologie nahe und liefert wichtige Anhaltspunkte für die Entwicklung bildgebender Biomarker zur Detektion früher Krankheitsstadien.

Zudem gibt es überzeugende Argumente für eine genetische Prädispositionen durch H1-Haplotypen am Tau-Genlocus *MAPT* als Hauptrisikofaktor für PSP und CBD. Bei der PSP wurden zudem in Genom-weiten Assoziationsstudien mögliche Genveränderungen in den Loci *MOBP* , STX6, EIF2AK3 und *C4A* beschrieben 7 8 . Diese Befunde verweisen auf axonale Myelinisierung, Stress am endoplasmatischen Retikulum und synaptische Vesikelwege als krankheitsrelevante Mechanismen.

## Klinische Manifestationen

Die klinische Präsentationen der PSP und CBD sind vielseitig und überschneiden sich gerade in frühen Krankheitsstadien mit anderen neurodegenerativen Erkrankungen wie der AK oder weiteren Parkinson-Syndromen wie der Parkinson Krankheit (PK) oder der Multisystematrophie (MSA), was die klinische Differenzialdiagnose herausfordernd macht 3 4 9 .

Die PSP wurde erstmals 1963 von Steele, Richardson und Olszewski beschrieben, die dabei den bis heute häufigsten klinischen Phänotyp, Richardson-Syndrom (RS) identifizierten. Dieses ist definiert durch ein axial betontes hypokinetisch-rigides Syndrom mit frühzeitig im Krankheitsverlauf auftretender Sturzneigung infolge gestörter posturaler Reflexe, sowie durch eine supranukleäre vertikale Blickparese, die der Erkrankung ihren Namen gab. Das klassische PSP-RS ist jedoch nur eine von vielen möglichen klinischen Manifestationen der PSP, die in etwa 25% der PSP-Fälle auftritt. Die PSP kann sich im frühen klinischen Verlauf aber auch mit frontaler Verhaltens- und Exekutivstörung, Apathie, Pseudobulbärsymptomatik (z. B. Dysarthrie/Dysphagie), Sakkadenverlangsamung oder Doppelbildern, einem Parkinson-Syndrom, Gang-Freezing, oder mit dem klinischen Bild eines CBS manifestieren. Diese Varianten konvergieren im längerfristigen Verlauf aber häufig zum Vollbild eines RS-Phänotyp 2 .

Die CBD wurde erstmals 1967 von Rebeiz et al. beschrieben und als corticodentatonigrale Degeneration mit neuronaler Achromasie bezeichnet 10 . Seitdem hat sich unser Wissen über die klinischen Merkmale und die zugrunde liegende Tau-Pathologie enorm erweitert. Bei der CBD liegt am häufigsten, etwa in einem Drittel der Fälle, das klinische Erscheinungsbild eines CBS vor 3 . Das CBS besteht aus zumeist asymmetrischen Störung der parietal-kortikalen (=„cortico“) und extrapyramidal-motorischen (=„basalganglionär“) Funktionen. Die kortikalen Symptome beinhalten das kortikal-sensorische Defizit, Extremitäten- oder Sprechapraxie und das „Alien limb“ Phänomen, während Dystonie, Myoklonus, Rigor und Akinese mögliche motorische Symptome des CBS darstellen. Die motorischen Symptome Rigor und Akinese des CBS können zu Beginn bei asymmetrischer Präsentation der PK ähnlich sein. Jedoch besteht in der Regel kein oder nur geringes Ansprechen auf eine dopaminerge Therapie. Unter den für die Diagnosestellung relevanten kortikalen Symptomen ist die orobukkale Apraxie charakterisiert durch eine stockende, erschwerte Sprachproduktion mit verzerrtem Artikulationsverhalten. Die zumeist ideomotorische Extremitätenapraxie kann sowohl obere als auch untere Gliedmaßen betreffen. Ein kortikal-sensorisches Defizit manifestiert sich als Unfähigkeit, Objekte, zum Beispiel Stift oder Schlüssel, taktil zu identifizieren bei intaker primärer (peripherer und spinaler) Wahrnehmungsfähigkeit. Das Alien-Limb-Phänomen beschreibt, dass eine betroffene Extremität nicht mehr als Teil des eigenen Körpers empfunden wird und quasi autonome, vom Patienten nicht bewusst initiierte Bewegungen vollführt. Die erkrankte Extremität wird von Patienten häufig als funktionslos und in seltenen Fällen als störend empfunden, da unwillkürliche Bewegungen zielgerichtete Handlungen beeinträchtigen können. Bei der CBD kommen neben dem CBS seltener Sprachstörungen (agrammatische primär progrediente Aphasie), frontale dysexekutive und behaviorale Syndrome oder RS-ähnliche Syndrome vor. Bei mehr als der Hälfte der Patienten liegt im fortgeschrittenen Krankheitsverlauf eine dementielle Entwicklung vor, jedoch bleibt das semantische Gedächtnis in der Regel erhalten.

Neuropathologisch kann dem klinischen Syndrom CBS oft eine andere Pathologie als die CBD zugrundeliegen. In je etwa einem Viertel der Fälle findet sich neuropathologisch eine PSP oder eine AK, oder seltener eine Synuclein- oder TDP-43 Proteinopathie 11 . Deshalb wird der histopathologisch definierte Begriff CBD heute nicht mehr zur Beschreibung des klinischen Erscheinungsbilds genutzt, sondern nur noch als neuropathologische Diagnose.

Im Gegensatz zu Synucleinopathien wie PK oder MSA sind REM-Schlafverhaltensstörungen, Restless-Legs-Syndrom und Atemregulationsstörungen bei Patienten mit PSP und CBD selten, da die für diese Symptome relevanten pontinen REM-Atonie-Kerne sowie hypothalamisch-dopaminerge Netzwerke in primären Tauopathien weit weniger betroffen sind 12 . Auch Halluzinationen und Psychosen finden sich im Unterschied zur Demenz mit Lewy Körperchen bei PSP und CBD nicht 2 3 . Im fortgeschrittenen Krankheitsstadium kommt es sowohl bei PSP als auch CBD durch die Ausbildung eines schweren hypokinetisch-rigiden Syndroms meist zu traumatisierenden Stürzen und Immobilität und durch Dysphagie zu Aspirationspneumonien.

## Aktuelle Diagnosekriterien

Da es für 4R-Tauopathien bislang keine validierten, in der klinischen Routine verfügbaren Biomarker gibt, die die Erkrankungen sensitiv und spezifisch diagnostizieren würden, beruhen die aktuellen Diagnosekriterien auf dem Nachweis der charakteristischen Syndrome und versuchen dabei der klinischen Variabilität der 4R-Tauopathien Rechnung zu tragen 2 3 .

### Aktuelle Diagnosekriterien der PSP

Die Diagnosekriterien der Movement Disorders Society für PSP setzen eine fortschreitende Erkrankung mit einem Erkrankungsalter von 40 Jahren oder älter voraus, die in erster Linie sporadisch auftritt, aber auch sehr seltene pathologische *MAPT* -Genvarianten umfassen kann 2 . Die heterogenen klinischen Varianten der PSP werden nach dem vorherrschenden klinischen Phänotyp benannt:

- PSP mit Richardson’s Syndrom, d. h. Haltungsinstabilität mit Fallneigung und Augenbewegungsstörungen (PSP-RS)
- PSP mit prädominanter Störung der **O** kulo **m** otorik (PSP-OM),
- PSP mit prädominanter **p** osturaler **I** nstabilität (PSP-PI),
- PSP mit prädominanter **P** arkinson-Symptomatik (PSP-P),
- PSP mit **p** rogredientem **G** ang **f** reezing (PSP-PGF),
- PSP mit **f** rontalem kognitiven und Verhaltens-Syndrom (PSP-F),
- PSP mit prädominanter Sprech-/Sprachstörung (engl.; **s** peech/ **l** anguage) einschließlich der nicht-flüssigen/agrammatikalischen Variante der primär-progredienten Aphasie und/oder der Sprechapraxie (PSP-SL),
- PSP mit prädominantem **k** ortiko **b** asalen **S** yndrom (PSP-CBS)

**Abb. 2:**
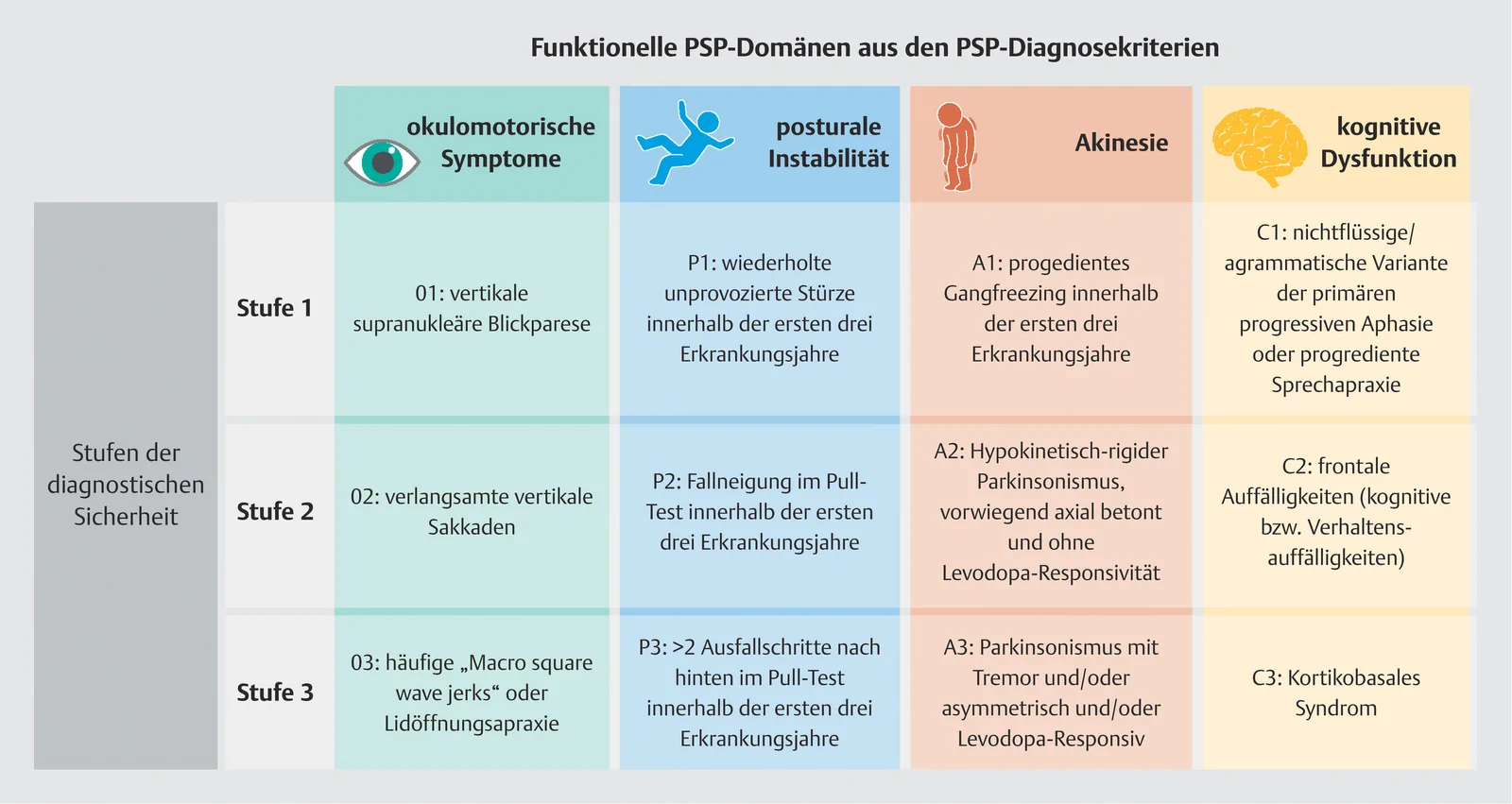
Die funktionellen Domänen der Progressiven Supranukleären Blickparese (PSP) umfassen nach den PSP-Diagnosekriterien der Movement Disorders Society 3 folgende Störungen: Okulomotorische Symptome, posturale Instabilität, Akinesie und kognitive Dysfunktion („OPAC“). Abhängig von der klinischen Ausprägung der Symptome in den einzelnen Domänen werden drei Stufen diagnostischer Sicherheit unterschieden, deren Grad mit dem Vorliegen spezifischer für die PSP charakteristischer klinischer Merkmale zunimmt. Stufe 1 hat die höchste klinische diagnostische Sicherheit.

Es wurden verschiedene funktionelle Domänen nach klinischer Ausprägung der Symptome definiert, anhand derer die Diagnosestellung erfolgen soll ( Abb. 2 ). Diese umfassen die sogenannten **OPAC** -Domänen „ **Okulomotorische Symptome** ”, „ **P** osturale Instabilität”, „ **A** kinesie” und „Kognitive Dysfunktion“ (engl; „ **C** ognitive Dysfunction“).

Abhängig von der klinischen Ausprägung der Symptome in den einzelnen Domänen werden drei Stufen diagnostischer Sicherheit unterschieden, deren Grad mit dem Vorliegen spezifischer für die PSP charakteristischer klinischer Merkmale zunimmt. Die Symptome der Stufe 1 besitzen die höchste Spezifität für PSP. Dazu zählen die charakteristische supranukleäre Blickparese, wiederholte unprovozierte Stürze oder ein fortschreitendes Gang-Freezing innerhalb der ersten drei Krankheitsjahre sowie eine progrediente Aphasie beziehungsweise Sprechapraxie. Abhängig von der Kombination der Symptomdomänen lässt sich eine wahrscheinliche oder mögliche PSP diagnostizieren beziehungsweise ein für PSP suggestives Syndrom beschreiben. Der neuropathologische Nachweis post mortem gilt weiterhin als Goldstandard der Diagnosestellung und definiert die definitive PSP im Sinne der höchsten diagnostische Sicherheit. Tab. 1 zeigt die Einstufung der diagnostischen Sicherheit für die verschiedenen PSP-Varianten.

**Table:** **Tab. 1** Diagnosesicherheit und PSP-Phänotypen nach den PSP-Diagnosekriterien der Movement Disorders Society 3 .

Diagnosesicherheit	Symptomatik	PSP-Phänotypen	Vorherrschende Lokalisation der Hirnatrophie und Pathologie
Voraussetzungen	Sporadische Erkrankungsform oder selten MAPT-Mutation nachweisbar langsam progredienter Verlauf Symptombeginn ab dem 40. Lebensjahr Ausschlusskriterien nicht erfüllt		
Wahrscheinliche PSP	(O1 oder O2)+(P1 oder P2)	PSP-RS	Hirnstamm, Basalganglien
	(O1 oder O2)+A1	PSP-PGF	Hirnstamm
	(O1 oder O2)+(A2 oder A3)	PSP-P	Hirnstamm, Basalganglien
	(O1 oder O2)+C2	PSP-F	Kortikal
Mögliche PSP	O1	PSP-OM	Hirnstamm
	O2+P3	PSP-RS	Hirnstamm,
	A1	PSP-PGF	Hirnstamm
	(O1 oder O2)+C1	PSP-SL	Kortikal
	(O1 oder O2)+C2	PSP-F	Kortikal
	(O1 oder O2)+C3	PSP-CBS	Kortikal
Suggestiv für PSP	O2 oder O3	PSP-OM	Hirnstamm
	P1 oder P2	PSP-PI	Hirnstamm
	O3+(P2 oder P3)	PSP-RS	Hirnstamm
	(A2 oder A3)+(O3, P1, P2, C1, C2, oder weitere klinische Anhaltspunkte)	PSP-P	Hirnstamm, Basalganglien
	C1	PSP-SL	Kortikal
	C2+(O3 oder P3)	PSP-F	Kortikal
	C3	PSP-CBS	Basalganglien, kortikal

Zusätzliche klinische Anhaltspunkte für eine PSP-Verdachtsdiagnose können eine Levodopa-Resistenz, eine hypokinetisch-spastische Dysarthrie, Dysphagie sowie eine ausgeprägte Photophobie sein. Ausschlusskriterien beinhalten AK-typische episodische Gedächtnisstörungen, ausgeprägte Dysautonomie oder Ataxie, Halluzinationen, kognitive Schwankungen, Affektionen des zweiten Motoneurons, (Autoimmun-) Enzephalitis in der Anamnese und plötzliches Auftreten oder schubweises Fortschreiten. Bildgebende Ausschlusskriterien sind schwere Leukoenzephalopathie, Hydrozephalus, schwerer Schlaganfall oder andere fokale Hirnläsionen.

Durch das bewusste Berücksichtigen des erweiterten phänotypischen Spektrums von PSP sind die aktuellen PSP-Kriterien sensitiver hinsichtlich der PSP-Diagnose. Um die Zuordnung zu den einzelnen PSP-Phänotypen zu erleichtern, wurden die Multiple Allocations eXtinction (MAX)-Regeln entwickelt. Diese Regeln basieren auf der diagnostischen Sicherheit für eine PSP und sind gewichtet nach dem bei Krankheitsbeginn vorherrschenden Phänotyp 13 . Die meisten Patienten schreiten innerhalb der ersten Jahre (im Durchschnitt 3,6 Jahre) von einer suggestiven oder möglichen PSP zu einer wahrscheinlichen PSP fort 14 .

### Diagnosekriterien der CBD

Die revidierten CBD-Kriterien aus dem Jahr 2013 3 definieren eine wahrscheinliche und mögliche zugrunde liegende CBD-Pathologie. Sie erfordern eine sporadische, progrediente Erkrankung über mindestens ein Jahr (u. a. zum Ausschluss rasch progredienter Erkrankungen wie der Creutzfeldt-Jakob-Krankheit) und den Ausschluss einer AK. In den CBD-Kriterien wurden vier primäre Phänotypen identifiziert, darunter CBS, frontales Verhaltens- und Raumverarbeitungs-Syndrom, nicht-flüssige oder agrammatische primär progrediente Aphasie und ein PSP-RS-Syndrom mit supranukleärer Blickparese, posturaler Instabilität und Akinese. Es wird beim häufigsten Phänotyp CBS eine Kombination aus kortikalen und subkortikalen Defiziten definiert (siehe Tab. 2 ). In den PSP-Kriterien 2 ist das CBS gemäß den OPAC-Kriterien innerhalb der funktionellen Domäne ‚Kognitive Dysfunktion‘ verortet. Demnach kann bei einem Patienten mit vertikaler Blickparese oder verlangsamten vertikalen Sakkaden in Kombination mit CBS-Symptomen die Diagnose einer PSP-CBS gestellt werden (vgl. *Aktuelle Diagnosekriterien der PSP* ). Diese Diagnose lässt sich unter Berücksichtigung der wahrscheinlichen neuropathologischen Grundlage als ‚wahrscheinliche 4R-Tauopathie‘ klassifizieren. Dies ist insofern sinnvoll, als nach Ausschluss einer Alzheimer-Pathologie am wahrscheinlichsten eine PSP- oder eine CBD-Pathologie in Betracht kommt.

**Table:** **Tab. 2** Diagnosekriterien für das CBS 4 .

Diagnose	Klinische Zeichen
Voraussetzungen	Sporadische, progrediente Erkrankung Krankheitsdauer mindestens 1 Jahr Kein Nachweis einer Alzheimer Krankheit
Wahrscheinliches CBS	Alter≥50 Jahre Asymmetrische Präsentation mit jeweils≥2 Symptomen aus kortikalen und subkortikalen Kriterien: Subkortikale Kriterien: Extremitätenrigor oder -akinese Extremitätendystonie Extremitätenmyoklonus Kortikale Kriterien: Orobukkale oder Extremitätenapraxie Kortikalsensorisches Defizit Alien-Limb-Phänomen
Mögliches CBS	Keine Altersgrenze Asymmetrische oder symmetrische Präsentation mit jeweils≥1 Symptom aus kortikalen und subkortikalen Kriterien

## Diagnostische Zusatzuntersuchungen

### Klinische Biomarker

**Abb. 3:**
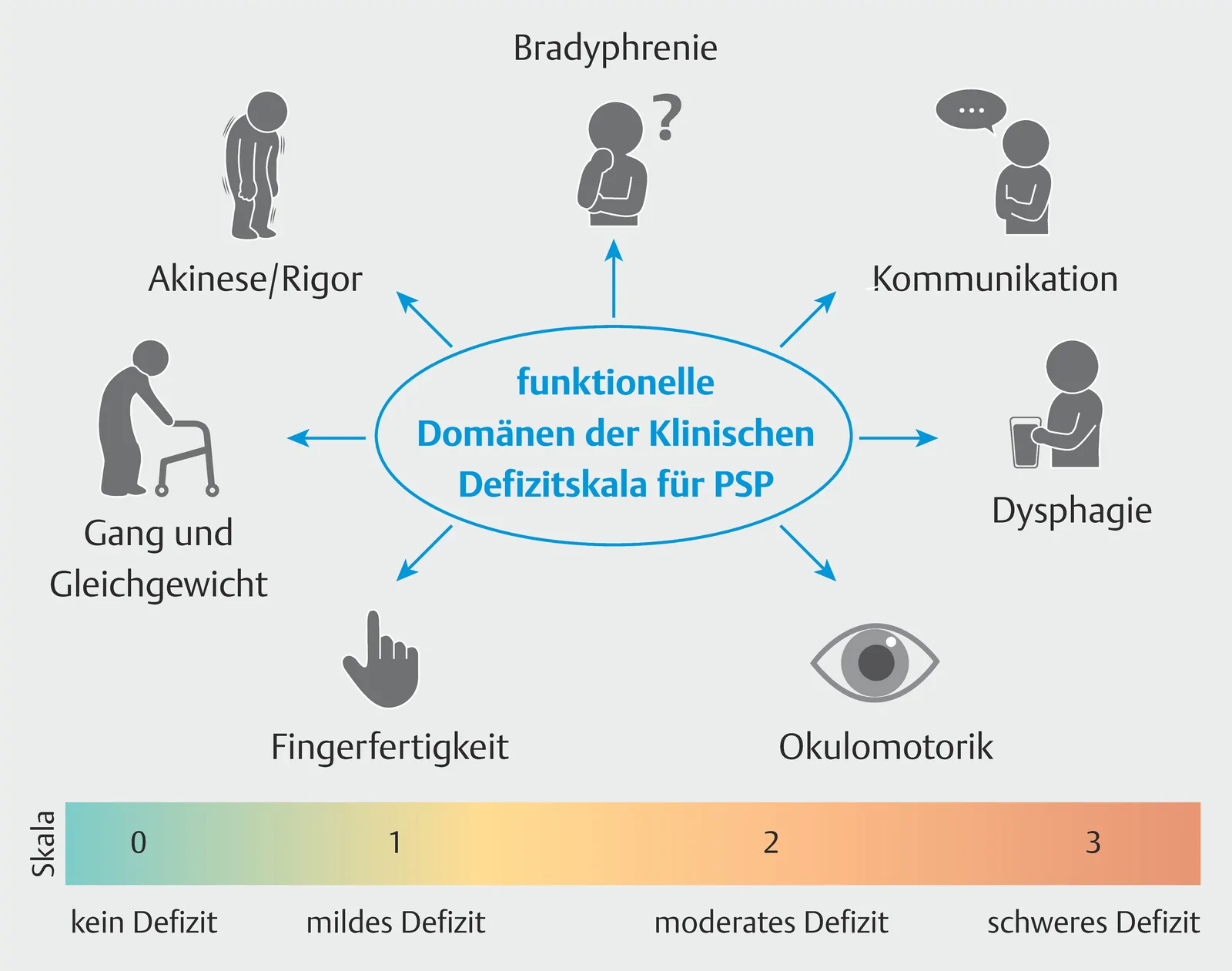
In der klinischen Defizitskala für Progressive Supranukleäre Blickparese (engl. PSP-Clinical Deficits Scale, PSP-CDS) der Movement Disorders Society werden die funktionellen Domänen Akinesie-Rigor, Bradyphrenie, Kommunikation, Dysphagie, Okulomotorik, Fingerfertigkeit und Gang und Gleichgewicht zwischen 0 (kein Defizit) und 3 (schweres Defizit) erfasst 17 .

Voraussetzung für die klinische Diagnose ist eine detaillierte Anamnese und neurologische sowie neuropsychologische Untersuchung. Nützliche Skalen zur Bewertung der Krankheitsschwere sind unter anderem die PSP Rating Scale (PSPRS) 15 sowie die PSP-Quality of Life Scale 16 , die die Lebensqualität misst. Beide weisen eine hohe Interrater-Reliabilität auf und lassen sich leicht in der Praxis anwenden. Für die klinische Routine zur Evaluation der alltäglichen Einschränkungen eignet sich zudem die Klinische Defizitskala für PSP (engl. PSP-Clinical Deficits Scale, PSP-CDS) 17 . In der PSP-CDS werden die funktionellen Domänen Akinesie-Rigor, Bradyphrenie, Kommunikation, Dysphagie, Okulomotorik, Fingerfertigkeit und Gang und Gleichgewicht zwischen 0 (kein Defizit) und 3 (schweres Defizit) bewertet ( Abb. 3 ). Die PSP-CDS ist zur Anwendung bei allen PSP-Phänotypen, einschließlich CBS, geeignet. Vorteil ist eine schnelle Durchführbarkeit am Patientenbett.

### Biomarker der Neurodegeneration

Ergänzend zur Klinik können bildgebende Befunde zur Darstellung der neurodegenerativen Prozesse die diagnostische Einschätzung stützen, insbesondere eine ausgeprägte Mittelhirnatrophie oder asymmetrische frontoparietale Atrophie im MRT, ein Hypometabolismus in der [ ^18^ F]DG-PET in den selben Regionen, sowie der Nachweis einer postsynaptischen striatalen dopaminergen Degeneration in der [ _123_ I]IBZM-SPECT or [ ^18^ F]-DMFP-PET 2 46 . Allerdings ist eine SPECT-Untersuchung bei 4R-Tauopathien selten indiziert. Die Befunde sind zwar häufig abnormal, jedoch unspezifisch, da vergleichbare Veränderungen auch bei anderen Parkinson-Syndromen auftreten. Die [ ^18^ F]DG-PET kann hingegen diagnostisch wertvolle Hinweise auf den zugrunde liegenden neurodegenerativen Prozessen liefern, bildet jedoch nicht die spezifische Tau-Pathologie der 4R-Tauopathien ab.

### Biomarker der Proteinpathologie

Zum differenzialdiagnostischen Ausschluss einer Alzheimer-Erkrankung wird bei CBS vor der Diagnosestellung typischerweise eine Liquoruntersuchung oder eine β-Amyloid-PET gemäß den AK-Diagnosekriterien durchgeführt 18 . Die *in vivo* Identifizierung von 4R-Tau-Pathologie im Gehirn mittels PET erfolgt derzeit primär im Forschungsbereich (siehe Kapitel *Aktuelle Forschungsschwerpunkte* ).

### Genetische Untersuchung

In den PSP-Kriterien wird empfohlen, eine genetische Beratung und Untersuchung durchzuführen, wenn mindestens ein Verwandter ersten oder zweiten Grades ein PSP-ähnliches Syndrom oder bekannte genetische Auffälligkeiten wie *MAPT* -Mutationen aufweist.

## Therapie

### Symptomatisch

Obwohl noch keine Heilung oder krankheitsmodifizierende Behandlung für diese Erkrankungen existieren, gibt es Strategien zur Verbesserung der Symptome und der Lebensqualität 19 . Die klinische Versorgung sollte idealerweise die Unterstützung durch ein multidisziplinäres Team umfassen, in dessen Mittelpunkt die betroffene Person und die Angehörigen stehen 20 . Eine Zusammenfassung ist in Tab. 3 aufgeführt.

**Table:** **Tab. 3** Symptomatische Therapien und Interventionen.

Wirkstoff/Intervention	Zielsymptome	Wirkmechanismus	Wichtige Nebenwirkungen/Hinweise
Physiotherapie	Mobilität, Stürze	Funktionserhalt, Gang- und Gleichgewichtstraining	Regelmäßige lebensbegleitende Maßnahme
Ergotherapie	Alltagsfunktionen, Akinese, Dystonie, Apraxie	Training alltagsrelevanter Fähigkeiten	Regelmäßige lebensbegleitende Maßnahme
Logopädie	Sprache, Sprechen und Schlucken	Verbesserung von Kommunikation und Schluckfunktion, Verhinderung von Aspiration, Diagnostik und Anpassung der Ernährung	Frühzeitig beginnen, Regelmäßige lebensbegleitende Maßnahme, perkutane gastroskopische Gastrostomie (PEG)-Anlage kritisch nach Patientenwille prüfen
Hilfsmittel (Stöcke, Rollator, Rollstuhl)	Stürze und Folgeschäden	Mechanische Stabilisierung	Einsatz bei fortgeschrittener Erkrankung ggf. erforderlich
Palliative Versorgung	Lebensqualität, Betreuung	Unterstützung bei Planung und Entscheidungen und Vorsorgevollmacht	Einbindung und Aufklärung frühzeitig sinnvoll
Augenhilfen (Prismenbrillen, Sonnenbrille, befeuchtende Augentropfen etc.)	Diplopie, Lichtempfindlichkeit, trockene Augen	Optische Unterstützung	Symptomorientiert, keine Empfehlung zu Augenmuskeloperationen
Levodopa/Benserazid oder Levodopa/Carbidopa	Akinese und Rigor (insbesondere bei früher PSP-P)	Dopaminerg	Wirksamkeit begrenzt; Testung über mindestens einen Monat in einer Dosierung von 1000mg täglich hinsichtlich Wirksamkeit, keine Empfehlung für Dopaminagonisten
Amantadin	Gang, Dysarthrie	Glutamaterg/dopaminmodulierend	Bis 600 mg/Tag; mögliche Unruhe, Halluzinationen
Botulinumtoxin A	Dystonie, Lidöffnungsapraxie, Sialorrhoe	Blockade neuromuskulärer Übertragung	Anwendung alle drei Monate intramuskulär, intraglanduläre Injektionen bei Sialorrhoe nur durch erfahrene Anwender, passagere Verschlechterung einer Dysphagie möglich
Antidepressiva (SSRI/SNRI)	Depression, Angst, Affektstörung	Serotonerge Modulation	Anticholinerge Medikation vermeiden, Apathie meist therapieresistent
Clonazepam	Myoklonien	GABAerg	Sedierung, Sturzgefahr
Coenzym Q10	Kognition, Motorik	Mitochondriale Unterstützung	Nicht erstattungsfähig; Nutzen abwägen
Cannabidiol/Medizinisches Cannabis	Diverse Symptome	Unklar	Keine Studienevidenz bei 4R-Tauopathien
Cholinesterase-Hemmer	Kognition	Steigerung von Acetylcholin	Einzelfallentscheidung, keine generelle Empfehlung aufgrund des Nebenwirkungsprofils

Die lebensbegleitende, regelmäßige Durchführung von Physiotherapie erhält Funktion und Mobilität, reduziert Stürze und unterstützt die Unabhängigkeit. Im Verlauf kann bei häufigen Stürzen ein Gehstock, ein Rollator oder ein Rollstuhl erforderlich sein. Die Ergotherapie fokussiert auf die Verbesserung bei Alltagsaktivitäten, während Sprachtherapie Sprechen, Sprache und Schlucken verbessert und frühzeitig begonnen und nachhaltig durchgeführt werden soll. Bei fortgeschrittener Dysarthrie sind alternative Kommunikationsmöglichkeiten, z. B. Tablets oder Worttafeln, wichtig. Dysphagie ist eine Hauptursache für Morbidität und Mortalität, weshalb regelmäßige Schluckuntersuchungen durchgeführt werden sollten. Das Anlegen einer dauerhaften perkutanen Magensonde sollte nur unter Berücksichtigung von Patientenwillen, Pflegezielen und Lebensqualität kritisch evaluiert werden. Sozialarbeiter und Spezialisten für palliative Versorgung unterstützen betroffene Personen und Angehörige bei Pflegeplanung, Patientenverfügungen und Entscheidungen zum Lebensende 20 .

Die medikamentöse Behandlung bietet meist nur begrenzten Nutzen, der mit Krankheitsfortschreiten abnimmt. Levodopa kann bei PSP mit akinetisch-rigider Symptomatik im Frühstadium bei manchen Patienten teilweise wirken, während PSP-PGF und andere Varianten meist nur kaum auf Levodopa ansprechen. Bei Patienten mit Parkinson-Symptomatik sollte ein Therapieversuch mit Levodopa (plus Benserazid oder Carbidopa) mit einer schrittweisen Dosistitration nach Wirksamkeit auf bis zu 1000 mg/Tag durchgeführt werden 2 . Bei guter Verträglichkeit sollte Levodopa über einen Zeitraum von mindestens einem Monat eingenommen werden, um eine Verbesserung beurteilen zu können. Dopaminagonisten werden bei 4R-Tauopathien aufgrund des ungünstigeren Nebenwirkungsprofils nicht empfohlen 20 . Amantadin bis zu einer Tagesdosis von bis zu 600 mg zeigt potenzielle positive Effekte auf Gang und Dysarthrie 21 . Anticholinergika sollten wegen der Gefahr der Zunahme von Verwirrtheit und Benommenheit vermieden werden 20 . Injektionen von Botulinumtoxin A können bei schmerzhafter Dystonie, Lidöffnungsapraxie oder Sialorrhoe von erfahrenden Behandlern eingesetzt werden 22 . Augenbefeuchtungsmittel, Prismenbrillen oder Sonnenbrillen unterstützen bei okulomotorischen Defiziten (Doppelbilder), trockene Augen und Photophobie. Cholinesterasehemmer haben bei den kognitiven Störungen der 4R-Tauopathien keinen nachgewiesenen Nutzen und können Gang und Dysphagie verschlechtern 23 . Antidepressiva aus der Gruppe der selektiven Serotonin-Wiederaufnahmehemmern oder Serotonin-Noradrenalin-Wiederaufnahmeemmern können ggf. Symptome wie Depression, Angst, Reizbarkeit oder eine pseudobulbäre Affektstörung verbessern 20 . Die Apathie spricht meist nicht auf diese Medikamente an. Benzodiazepine als Anxiolytika sollten sehr zurückhaltend eingesetzt werden, da sie bei PSP-RS mit schnellerem Krankheitsverlauf assoziiert zu sein scheinen und die Fallneigung verstärken können 24 25 . Bei Myoklonien bei CBS kann eine Therapie mit Clonazepam hingegen sinnvoll sein. Ein positiver Nutzen von Coenzym Q10 auf kognitive und motorische Symptome war in Studien nachzuweisen 26 27 , werden aber als Nahrungsergänzungsmittel nicht von den Krankenkassen erstattet und müssen von Patienten daher selbst bezahlt werden. Deren therapeutischer Nutzen muss daher im Einzelfall sorgfältig abgewogen werden. Zur symptomatischen Wirkung von Cannabidiol und medizinischem Cannabis gibt es noch keine aussagekräftigen Studien bei betroffenen Personen mit 4R-Tauopathien.

### Krankheitsmodifizierend

Aktuell gibt es keine zugelassenen krankheitsmodifizierenden Therapien der 4R-Tauopathien. Auf Forschungsansätze zur Therapieentwicklung wird im Kapitel *Therapieentwicklung* näher eingegangen.

## Aktuelle Forschungsschwerpunkte

Die Forschung zu PSP und CBD hat in den letzten Jahren erheblich an Dynamik gewonnen, mit dem Ziel, ein vertieftes Verständnis der Krankheitsmechanismen zu erlangen, diagnostische Verfahren zu verbessern und neue therapeutische Ansätze zu entwickeln. Im Zentrum steht die Aufklärung der komplexen Neurobiologie dieser primären 4R-Tauopathien, die durch pathologische Aggregation von Tau-Protein und daraus resultierende neuronale Dysfunktion gekennzeichnet sind.

### Ätiopathogenese

Neuropathologische und molekulare Studien konzentrieren sich auf die Identifikation pathogener Tau-Spezies und deren posttranslationalen Modifikationen (PTMs), die Aggregation und zelluläre Ausbreitung fördern 5 . Neuere Modelle deuten darauf hin, dass spezifische 4R-Tau-Konformationen neurotoxisch wirken und über prionähnliche Mechanismen interzellulär übertragen werden. Ziel aktueller Arbeiten ist die molekulare Charakterisierung dieser Spezies sowie die Untersuchung der zellulären Ausbreitungswege, etwa über synaptische Transmission oder Exosomen.

Parallel dazu werden genetische und epigenetische Risikofaktoren erforscht. Varianten im *MAPT* -Gen (z. B. H1-Haplotyp) gelten als die wichtigsten genetischen Risikofaktoren für PSP und CBD. Darüber hinaus wird die Rolle von DNA-Methylierungsmustern, RNA-Editing und regulatorischen nichtkodierenden RNAs (miRNA, lncRNA) untersucht, die die Tau-Expression und Neuroinflammation modulieren können. Epidemiologische Studien zielen auf potenzielle Umweltfaktoren wie Pestizidexposition oder metabolische Stressoren ab, die in einer Gen-Umwelt-Interaktion zur Krankheitsmanifestation beitragen könnten 28 29 .

### Krankheitsmodelle

Zur Untersuchung dieser Mechanismen werden zunehmend humanisierte Krankheitsmodelle eingesetzt. iPSC-basierte Neurone und dreidimensionale Gehirnorganoide ermöglichen die Analyse tauopathischer Prozesse in patientenspezifischen Zellkontexten. Ergänzend liefern Tiermodelle mit 4R-Tau-Überexpression durch induzierte Genmutationen wichtige Erkenntnisse zur Pathogenese, Krankheitsprogression und Therapieentwicklung 29 .

### Biomarker

Ein zentraler Forschungsschwerpunkt liegt in der Entwicklung und Validierung biochemischer Biomarker für Diagnose und Verlaufsbeurteilung. Neurofilament-Leichtkette (NfL) gilt als unspezifischer Marker der Neurodegeneration und korreliert mit Krankheitsprogression und Prognose 30 31 . Im Fokus stehen zudem krankheitsspezifische Tau-basierte Marker, darunter phosphorylierte und fragmentierte Tau-Formen sowie „seed amplification assays“ zur Detektion pathogener Tau-Spezies 32 . Moderne Proteomik- und Multi-Omics-Ansätze ermöglichen ein umfassendes Profiling von Liquor und Plasma, um krankheitsspezifische Muster zu identifizieren. Neben Liquor gewinnen leicht zugängliche Materialien wie Blut, Plasma und Hautbiopsien an Bedeutung, um die Translation in die klinische Routine zu erleichtern.

In der Bildgebungsforschung wird die diagnostische Aussagekraft bestehender Verfahren optimiert. MRT- und FDG-PET-basierte Algorithmen werden durch KI-gestützte, automatisierte Auswertungen ergänzt, um typische Muster wie Mittelhirnatrophie (PSP) zuverlässig zu identifizieren 33 . Tau-PET-Tracer zur Darstellung von Tau-Ablagerungen werden derzeit für die klinische Anwendung bei 4R-Tauopathien validiert 34 . Parallel dazu ermöglichen Inflammations-PET-Verfahren (z. B. TSPO- oder MAO-B-Liganden) die in-vivo-Darstellung von Mikroglia- und Astrozytenaktivierung, was neue Einblicke in neuroinflammatorische Mechanismen bietet 35 .

### Krankheitsmonitorierung und Outcome Measures

Für die klinische Forschung werden neue Outcome Measures entwickelt, die eine präzise und sensitive Verlaufsbeurteilung ermöglichen. Neben der Modified PSP Rating Scale (mPSPRS; 36 ), die auf klinischen Meilensteinen basiert, kommen digitale Assessments (z. B. Wearables, Video-Pull-Test) und Patient-Reported Outcomes wie Lebensqualitätsfragebögen 37 zum Einsatz. Ziel ist die Etablierung reliabler, schnell durchführbarer Instrumente mit hoher Interrater-Reliabilität.

### Frühdiagnose und Prädiktion

Ein weiteres Ziel ist die Frühdiagnose anhand spezifischer Biomarker bei klinisch suggestiven, aber noch unspezifischen Symptomen (entsprechend der Diagnose „suggestiv für PSP“). Künstliche Intelligenz, Sensoren und Smartphone-basierte Tools könnten zukünftig subtile motorische Veränderungen automatisch detektieren. Im Rahmen der Prädiktionsforschung werden multimodale Baseline-Daten genutzt, um den individuellen Krankheitsverlauf zu modellieren – ein entscheidender Schritt für personalisierte Therapieplanung und Studienrekrutierung. Eine frühere Diagnose der Krankheit könnte Behandlungen, insbesondere solche, die auf eine Krankheitsmodifikation abzielen, wirksamer machen. In Deutschland erheben aktuell insbesondere die Register ProPSP und Describe-PSP des deutschen Zentrums für Neurodegenerative Erkrankungen (DZNE) prospektive Daten 38 . Ziel dieser Studien ist es, die Progression motorischer, kognitiver und verhaltensbezogener Symptome systematisch zu erfassen, Komplikationen zu dokumentieren und typische Krankheitsverläufe zu charakterisieren.

### Therapieentwicklung

Die Therapieentwicklung fokussiert auf krankheitsmodifizierende und symptomatische Strategien (siehe Übersicht in Tab. 4 ). Da Tau-Pathologie als zentraler Krankheitsmechanismus von PSP und CBD gilt, verfolgen aktuelle krankheitsmodifizierende Therapiestudien unterschiedliche Strategien, um die pathologische Tau-Akkumulation, -Aggregation oder -Propagation gezielt zu beeinflussen 39 . So werden beispielsweise Antisense-Oligonukleotide (ASOs) getestet, die die Tau-Expression reduzieren 40 , sowie monoklonale Antikörper, die die interzelluläre Tau-Ausbreitung hemmen. Weitere Ansätze umfassen aktive Immunisierung 41 , PROTACs (kurz von *englisch* Proteolysis targeting chimera) zur gezielten intrazellulären Tau-Degradation 42 , oder Inhibitoren posttranslationaler Modifikationen wie O-GlcNAcase (OGA)-Inhibitoren 43 . Derzeit wird zudem die Wirkung von Glycerolphenylbutyrat, einer kurzkettigen Fettsäure mit mutmaßlich neuroprotektiven Effekten bei CBS untersucht. Die Modulation lysosomaler Funktionen stellt einen weiteren vielversprechenden therapeutischen Ansatz dar, da Störungen der lysosomalen Homöostase wesentlich zur Tau-Akkumulation, neuronalen Dysfunktion und neuroinflammatorischen Prozessen bei PSP und CBD beitragen 44 . Darüber hinaus werden antiinflammatorische Strategien **,** die auf die Modulation der Mikroglia-Aktivierung abzielen, als potenziell krankheitsmodifizierende Therapien erforscht.

**Table:** **Tab. 4** Auswahl an Therapieentwicklungen für PSP und CBD.

Therapieansatz	Beschreibung/Mechanismus	Beispiele/klinische Studien
Tau‑gerichtete Antisense-Oligonukleotides (ASOs)	Reduktion der Tau‑Expression	*NIO752* , Novartis Pharamceuticals (Clinicaltrials.gov: NCT04539041; laufend)
Monoklonale Anti‑Tau‑Antikörper	Hemmung der interzellulären Ausbreitung von Tau	*Bepranemab* , UCB Biopharma SRL, (Clinicaltrials.gov: NCT04185415, beendet, NCT04658199, geplant) und *Gosuranemab* , Biogen (Clinicaltrials.gov: NCT03068468, beendet)
Aktive Immunisierung	Spezifische Immunantwort gegen Tau	AADVac1, Axon Neuroscience (Clinicaltrials.gov: NCT07173803, geplant)
O-GlcNAcase (OGA) ‑ Inhibitoren	Modulation posttranslationaler Modifikationen	*FNP-223* , Ferrer International S.A. (Clinicaltrials.gov: NCT06355531, laufend)
Glycerolphenylbutyrat	Kurzkettige Fettsäure mit mutmaßlich neuroprotektiven Effekten	Prüfer-initiierte Studie PROFIL-CBS (Glinicaltrials.gov: NCT05983588, laufend)
Lysosomale Modulation	Kleines Molekül zur Stabilisierung des Progranulin–Prosaposin-Signalwegs	*AZP2006,* Alzprotect SAS (Clinicaltrials.gov: NCT04008355, beendet, Studie Phase 2b/3 geplant)
Proteolysis targeting chimera (PROTACs)	Gezielte intrazelluläre Tau‑Degradation	Präklinische Studien
Anti-inflammatorische Strategien	Modulation der Mikroglia‑Aktivierung	Präklinische Studien
Nicht‑pharmakologische/apparative Ansätze	Vestibuläre Stimulation zur Verbesserung von posturaler Stabilität, Robotik, Neurorehabilitation	z. B. Low-intensity vestibular noise stimulation

Parallel werden symptomatische Ansätze weiterentwickelt, die u. a. Haltungsstabilität, Akinese, Okulomotorik, Sprache und Apathie adressieren. Fortschritte in der nicht-pharmakologischen und apparategestützten Therapie – etwa vestibuläre Stimulation 45 , Robotik und Neurorehabilitation – ergänzen pharmakologische Entwicklungen.

Zentrale Herausforderungen bleiben die Optimierung von Studiendesigns, insbesondere für kleine, proof-of-concept-orientierte Studien mit robusten Endpunkten und Biomarkerintegration.

## Zusammenfassung

PSP und CBD sind primäre 4-Repeat-Tauopathien, die unterschiedliche neuropathologische Muster und klinische Phänotypen aufweisen. Beide Erkrankungen gehören zum Spektrum der atypischen Parkinsonsyndrome und zeigen eine ausgeprägte klinische Heterogenität, die die bei der diagnostischen Abgrenzung für behandelnde Ärztinnen und Ärzte eine Herausforderung darstellt, die aber mit den o.g. Diagnosekriterien der Movement Disorders Society lösbar ist. Diese neuen klinischen Kriterien berücksichtigen die klinische Variabilität und ermöglichen eine frühere und sensitivere Diagnosestellung. Eine differenzierte Beurteilung von Krankheitsanamnese, Verlauf und spezifischen klinischen Merkmalen ist weiterhin essenziell. Ergänzend unterstützen moderne diagnostische Instrumente, darunter strukturelle und funktionelle Bildgebung (MRT, PET) sowie Flüssigbiomarker, die frühe Identifikation der Erkrankung. Trotz dieser Fortschritte besteht weiterhin ein erheblicher Forschungsbedarf, insbesondere zur Validierung von Biomarkern für die objektive Frühdiagnose, Verlaufsdokumentation, Prognoseabschätzung und Therapieevaluierung, um zukünftig gezielte krankheitsmodifizierende Behandlungsstrategien entwickeln und einsetzen zu können.

## Fazit für die Praxis

PSP und CBD zeigen eine hohe klinische Variabilität, die die Diagnose erschwert. Die diagnostischen PSP-Kriterien der Movement Disorder Society sowie moderne Bildgebung und Biomarker ermöglichen jedoch eine frühere und sensitivere Diagnosestellung.

Eine Anbindung der betroffenen Personen an ein spezialisiertes Zentrum für Bewegungsstörungen sollte angestrebt werden, um eine optimale diagnostische Abklärung, interdisziplinäre Betreuung und Zugang zu aktuellen Therapiestudien zu gewährleisten.
